# The Evolution of Antimicrobial Resistance in *Acinetobacter baumannii* and New Strategies to Fight It

**DOI:** 10.3390/antibiotics14010085

**Published:** 2025-01-14

**Authors:** Viola Camilla Scoffone, Gabriele Trespidi, Giulia Barbieri, Arooba Arshad, Aygun Israyilova, Silvia Buroni

**Affiliations:** 1 Department of Biology and Biotechnology, University of Pavia, 27100 Pavia, Italy; viola.scoffone@unipv.it (V.C.S.); gabriele.trespidi@unipv.it (G.T.); giulia.barbieri@unipv.it (G.B.); arooba.arshad@unipv.it (A.A.); 2 Laboratory of Microbiology, Center of Excellence, Baku State University, AZ1148 Baku, Azerbaijan; aygunisrayilova@bsu.edu.az; 3 Department of Biomedical Materials by ICESCO, Baku State University, AZ1148 Baku, Azerbaijan

**Keywords:** *Acinetobacter baumannii*, drug resistance, new therapy

## Abstract

*Acinetobacter baumannii* is considered one of the prioritized ESKAPE microorganisms for the research and development of novel treatments by the World Health Organization, especially because of its remarkable persistence and drug resistance. In this review, we describe how this can be acquired by the enzymatic degradation of antibiotics, target site modification, altered membrane permeability, multidrug efflux pumps, and their ability to form biofilms. Also, the evolution of drug resistance in *A. baumannii*, which is mainly driven by mobile genetic elements, is reported, with particular reference to plasmid-associated resistance, resistance islands, and insertion sequences. Finally, an overview of existing, new, and alternative therapies is provided.

## 1. Introduction

Nowadays, drug resistance is considered one of the most concerning and developing challenges in the world [1,2]. The World Health Organization (WHO) claimed that annually, antibiotic-resistant microbes are responsible for more than a million infections, which cause at least 23,000 deaths in the USA, and it is expected that the number of fatalities will increase tenfold by 2050 [1,3]. According to a report by the WHO, *Acinetobacter baumannii* is considered one of the prioritized microorganisms for the research and development of novel treatments. The pathogen has been classified as a principal ESKAPE (*Enterococcus faecium*, *Staphylococcus aureus*, *Klebsiella pneumoniae*, *A. baumannii*, *Pseudomonas aeruginosa*, and *Enterobacter* spp.) organism that can show resistance against antimicrobial drugs with adverse reactions [3,4].

*A. baumannii,* the primary member of the *Acinetobacter baumannii–calcoaceticus* (Abc) complex, is a non-fermenting Gram-negative coccobacillus that is obligately aerobic and opportunistically pathogenic. It is linked to healthcare-related infections worldwide [5,6] and has emerged as a persistent infectious agent in both nosocomial and community settings globally [7]. It can also be isolated from a variety of non-hospital settings, including soil, water, animals, humans, food (especially raw veggies), and inanimate objects [8,9]. Because of their remarkable persistence, these bacteria have a distinct advantage for survival in unfavorable conditions like hospitals where disinfectants and antibiotics are widely applied [10,11]. Several studies have identified neonatal units and burn wards as the primary settings in which critically ill patients are more susceptible to infections, posing a significant risk in these environments because of the propensity to develop extensive drug resistance [12,13].

Many reports showed that drug-resistant strains of *A. baumannii* have emerged and developed extensively as a result of the growing prevalence of the use of β-lactam antibiotics [14]. Recently, carbapenems were recommended as an efficient therapy for multidrug-resistant (MDR) *A. baumannii* infections, but their extensive use has increased the frequency of carbapenem resistance as well [15]. Currently, polymyxins are the preferred antibiotics for MDR *A. baumannii* infections, despite initial hesitancy due to the associated systemic toxicities, including nephrotoxicity and neurotoxicity [16]. *A. baumannii* isolates that are resistant to antibiotics, such as carbapenems, cephalosporins, aminoglycosides, and fluoroquinolones, have been classified as extensively drug resistant (XDR). On the other hand, *A. baumannii*, which is resistant to polymyxins and tigecycline, is known as pan-drug resistant (PDR) [17]. Over the years, its rapid acquisition of antibiotic resistance has elevated it to a global health burden, causing approximately 7300 infections and 500 deaths per year [18]. A multicentre cross-sectional study showed that the prevalence of MDR *A. baumannii* rose from 79% to 98%, while XDR cases increased from 47% in the pre-COVID-19 era to 69% in the post-COVID-19 period [19]. Indeed, from 2012 to 2020, the European Centre for Disease Prevention and Control (ECDC) showed an increase of 3.4% in fluoroquinolones, aminoglycosides, and carbapenems-resistant strains (a rise of 11.3% in Italy only) [20].

*A. baumannii* isolates acquire resistance in various ways, for example, enzymatic degradation of antibiotics, target site modification, altered membrane permeability, multidrug efflux pumps, and biofilm formation [21,22]. Biofilm formation is an important virulence mechanism for bacteria. The development and maintenance of *A. baumannii* biofilms are influenced by different microbial characteristics such as adhesion, surface appendages, virulence genes, and resistance determinants, along with physicochemical factors like temperature, growth media, pH, and oxygen concentration [11,13]. Biofilms can easily form on the surfaces of medical devices or hospital equipment (artificial joints, ventilators, and urinary or intravascular catheters), and they create an opportunity for pathogens to enter the body. *A. baumannii* can infect individuals by penetrating their skin and airways, making hospitalized and vulnerable patients more susceptible to infections [23]. *A. baumannii*, which is associated with a 60% mortality rate in severe infections, is the predominant pathogen responsible for ventilator-associated pneumonia, catheter-associated bloodstream infections, urinary tract infections, and secondary meningitis [12,24,25]. It is particularly prevalent among high-risk populations, especially immunocompromised patients in intensive care units (ICUs), which account for 20% of infections worldwide [25]. In this case, hospitalized patients are at high risk for *Acinetobacter* infection because of the effective colonization of bacteria on the abiotic surfaces [26]. Recently, it was shown that clinical isolates of *A. baumannii* demonstrated a superior capacity to form biofilm on abiotic surfaces compared with wild-type isolates [12].

The genetic plasticity of *Acinetobacter* is a critical factor, enabling swift genetic mutations, rearrangements, and the integration of foreign determinants through mobile genetic elements. The genetic plasticity of *A. baumannii* results in significant heterogeneity among isolates, complicating its study as a distinct entity. Overall, mobile genetic elements have been reported as drivers of antimicrobial resistance evolution in *A. baumannii*. Plasmids, resistance islands, and insertion sequences are regarded as significant factors influencing bacterial genomes and, consequently, evolution.

This review provides a concise overview of the classical mechanisms of antibiotic resistance, including biofilm formation, the evolution of drug resistance in *A. baumannii*, and emerging treatments to cure infections.

## 2. Mechanisms of Drug Resistance in *A. baumannii*

### 2.1. Enzymatic Inactivation

*A. baumannii* produces several enzymes that degrade or modify antibiotics, rendering them ineffective. Among them, β-lactamases were divided into four classes (Table 1). Class A enzymes hydrolyze penicillin and include carbapenemases [27]. Class B metallo-β-lactamases (MBLs) can hydrolyze penicillins, cephalosporins, and carbapenems. Among the MBLs in *A. baumannii,* there are the New Delhi metallo-β-lactamase (NDM), the Verona integron-encoded metallo-β-lactamase, and the imipenemase. Strains producing these enzymes are often resistant to all the β-lactams except monobactams [28]. Class C, the chromosomally encoded AmpC β-lactamase, hydrolyzes cephalosporins, is not usually inhibited by clavulanic acid, and its expression is induced in the presence of β-lactams. These enzymes were classified as *Acinetobacter*-derived cephalosporinases, and their overexpression is caused by an insertion sequence (ISAba1). Many different variants have been described that confer resistance against penicillins, extended-spectrum cephalosporins, monobactams, and β-lactamase inhibitors [29]. Class D (OXA-type) oxacillinase enzymes, with a broader substrate profile, can hydrolyze carbapenems. Carbapenem-resistant *A. baumannii* (CRAB) is well-known for producing these enzymes, namely OXA-23, OXA-24/40, and OXA-58. Moreover, *A. baumannii* strains possess a chromosomally encoded OXA-51-like β-lactamase. OXA-type β-lactamases (especially OXA-23) have also been identified in cefiderocol-resistant *A. baumannii* [30]. As more than 400 OXA-type β-lactamases have been described, the quantity and variety of these enzymes represent a serious challenge in *A. baumannii* infection containment. In some cases, such as cefiderocol resistance, a combination of factors contribute to resistance, including the presence of β-lactamases (NDM-like enzymes), modification of the penicillin-binding proteins (target gene PBP-3), permeability defects associated with efflux pump overexpression, and reduced expression or mutation of genes involved in the ion transport [31].

Aminoglycoside-modifying enzymes (AMEs) include acetyltransferases, phosphotransferases, and nucleotidyltransferases inactivating aminoglycosides (e.g., gentamicin, amikacin) by acetylation, phosphorylation, or adenylation, preventing them from binding to their bacterial ribosomal target. Mutations in the aminoglycoside transferase AAC(6′)-Ib-Cr allow N-acetylation of two fluoroquinolones (ciprofloxacin and norfloxacin) [32]. Several reports showed clinical isolates with a match in genes coding for aminoglycoside modification enzymes *ant(3″)-I*, *aac(3)-I*, *aph(3′)-I*, *aac(6′)-Ib,* and *aph(3′)-IIb* [33]. Genes encoding AME enzymes are located on mobile genetic elements, facilitating the spread through bacterial populations [34].

### 2.2. Target Site Modification

Alterations in penicillin-binding protein (PBP) encoding genes, both modification or overexpression, reduce the binding affinity of β-lactam antibiotics for their targets. Modifications can occur by mutations in the genes encoding PBPs or by acquiring new PBP genes from other bacteria [35].

Modifications of the 16S rRNA component of the 30S ribosomal subunit, such as its methylation, are one of the most significant alterations causing the change of the binding site for aminoglycosides [36].

The structural modification of lipid A, usually by the addition of phosphoethanolamine (PEtN) and 4-amino-4-deoxy-L-arabinose (L-Ara4N), is the primary mechanism of colistin resistance in *A. baumannii*. When these groups are attached to lipid A, the lipopolysaccharide (LPS) negative charge is reduced, decreasing the binding efficacy of colistin [37]. A large number of *A. baumannii* colistin-resistant strains carried mutations in the genes encoding the PmrAB two-component regulatory system, upregulating the expression of the *pmrCAB* operon. In the activated state, PmrA regulates the expression of the *pmrC* gene, encoding a phosphoethanolamine transferase that catalyzes the addition of PEtN to lipid A [37]. Other studies showed that PmrA also regulates the N-acetylhexosamine deacetylase, involved in the deacetylation of β-galactosamine, thus modifying lipid A [37]. Recently, a plasmid-mediated resistance to polymyxin has been described in *A. baumannii*, which carries the mobile colistin resistance gene *mcr*, encoding a phosphoethanolamine transferase that adds PEtN to lipid A [38]. Insertion mutations into the *hns* gene alter the expression of more than 150 genes, among which is *eptA*, coding for a PEtN transferase homolog to PmrC, which confers colistin resistance [39].

Some *A. baumannii* strains completely lose the LPS due to mutations or altered expression of lipid A biosynthesis genes (*lpxA*, *lpxC,* and *lpxD*). This important modification deeply alters the outer membrane, completely removing the binding target of colistin [40]. Moreover, this mutation reduces the outer membrane’s negative charge and permeability, decreasing colistin effectiveness [41].

*A. baumannii* also shows mutations in *gyrA* and *parC* genes, coding for the DNA gyrase subunit and the topoisomerase IV subunit C, respectively, that confer direct fluoroquinolone resistance [42].

### 2.3. Altered Membrane Permeability

The reduction of membrane permeability impairs the activity of hydrophilic antibiotics, such as β-lactams, aminoglycosides, and tigecycline. Moreover, the alteration in the outer membrane modifies the entry of antibiotics and reduces their intracellular concentration.

Porin channel modifications, such as expression level alteration or structural changes, limit antibiotic uptake. In *A. baumannii*, the loss or downregulation of the OmpA and CarO porins (33–36 kDa) are associated with carbapenem resistance and help the passive diffusion of antibiotics [43]. Regarding the structural changes of the porin proteins, mutations in these genes can alter the size and the charge of the porins; in this case, the mutations arise from the selective pressure exerted by the intense clinical use of antibiotics [34].

Changes in the outer membrane lipids, such as modifications in LPSs, contribute to resistance against polymyxins (e.g., colistin). The overall charge and the hydrophobicity of the membrane can be altered, blocking the diffusion of molecules. In particular, lipid A can acquire structural modifications that alter the membrane barrier function, thanks to horizontal gene transfer of genes encoding enzymes able to alter its structure [44].

Moreover, *A. baumannii* is characterized by a dense, polysaccharide-rich capsule that functions as a physical barrier against antibiotics, in particular aminoglycosides and other antibiotics requiring interaction with the outer membrane [45].

### 2.4. Active Efflux

In *A. baumannii,* three resistance nodulation cell division (RND)-family efflux pumps, AdeABC, AdeFGH, and AdeIJK, and the multi-antimicrobial and toxic compound extrusion (MATE)-family of efflux pumps are overexpressed due to amino acid substitutions in their regulatory genes [46], inducing resistance to aminoglycoside, chloramphenicol, erythromycin, tetracycline, and tigecycline [43]. Furthermore, the plasmid-encoded *qepA* gene codifies a major facilitator superfamily (MFS) efflux pump that increases resistance to fluoroquinolones (particularly to ciprofloxacin) [47].

The most characterized efflux system is the AdeABC efflux pump, belonging to the RND family, which extrudes a wide variety of antibiotics, among which fluoroquinolones, tetracyclines, and chloramphenicol. AdeABC is composed of three proteins, with AdeB as the critical component that functions as the multi-drug transporter. In the two-component system, AdeR-AdeS controls the expression of this efflux pump, and mutations in the genes codifying these two proteins, such as A94V and S8A in *adeS* or P56S in *adeR,* lead to overexpression of the pump, increasing the resistance levels [43]. Moreover, the insertion of genetic elements, such as ISAba1 into *adeS*, has been demonstrated to increase *adeB* expression. Environmental pressure, such as exposure to sub-lethal doses of tigecycline, increases the expression of the efflux pump, allowing bacteria to survive in the presence of the antibiotic [48].

### 2.5. Biofilms and Antibiotic Resistance

*A. baumannii* biofilms contribute to persistence and multi-drug resistance. Due to its complex structure, composed of a matrix of polysaccharides, proteins, nucleic acids, and lipids, biofilms provide an environment that protects bacteria against antibiotics and immune system cells. The antibiotic dose necessary to eradicate biofilms can be up to 1000 times higher than the quantity needed to kill bacteria in planktonic growth [49]. Biofilm formation is controlled by numerous genes and environmental factors. Among the genes that play a critical role in cell adhesion, biofilm maturation, and structural stability, there are *bap* (biofilm-associated proteins), *ompA* (outer membrane protein A), *csuE* (part of the chaperone-usher pathway), and *pgaB* (involved in polysaccharide production).

Moreover, a complex quorum sensing system, AbaI/AbaR, analog of the LuxI/LuxR system, is involved in biofilm formation regulation [50] together with autoinducer-2, a universal signal molecule engaged in interspecies communication, that boosts biofilm formation.

## 3. Mobile Genetic Elements as Drivers of Antimicrobial Resistance Evolution in *A. baumannii*

Among the nine clonal lineages known so far, the globally spread *A. baumannii* Global Clone 1 (GC1) and Global Clone 2 (GC2) are key drivers of carbapenem-resistant *A. baumannii* outbreaks. Corresponding to sequence types ST1 and ST2, these clones are a major concern in persistent nosocomial infections. Their ability to acquire antimicrobial resistance is facilitated by horizontal gene transfer, mediated by mobile genetic elements (MGEs) such as plasmids, transposons, and integrons. These incorporate resistance genes into the genome, enhancing adaptability to antimicrobial pressures and contributing to persistent infections in healthcare settings [51].

Early isolates of GC1 and GC2 clones harbored resistance genes targeting early antibiotics such as tetracycline, sulfonamides, and certain aminoglycosides. Subsequent evolutionary events, driven by horizontal gene transfer, facilitated the emergence of strains resistant to modern antibiotics, including fluoroquinolones, third-generation cephalosporins, and carbapenems. These processes have led to substantial genetic diversity within each clonal complex, resulting in the identification of distinct lineages and sub-lineages [52,53].

### 3.1. Plasmid-Associated Resistance

The plasmids identified in *Acinetobacter* species play a key role in the spread of antibiotic resistance genes and are largely confined to this genus, as they do not appear to be stably maintained in other Gram-negative bacteria, particularly Enterobacterales [54].

The vast majority of *A. baumannii* strains carry at least one plasmid. An analysis of 813 complete plasmid sequences, classified using the *Acinetobacter* Plasmid Typing scheme based on the DNA sequence identity of the replication initiation genes (*rep*), led to the definition of three families: R1, R3, and RP. A fourth group lacking an identifiable Rep protein was classified as the “*rep*-less” group [55]. While R1-type plasmids encoding the Pfam01446 replication protein are not linked to antimicrobial resistance (AMR), various R3, RP, and *rep*-less plasmids were associated with the spread of carbapenem resistance genes [55].

R3 plasmids encoding Rep_3-type replication proteins (Pfam01051) represent the most diverse group of *A. baumannii* plasmids. They are carried by all major global clones, predominantly GC2 and GC1, and exhibit broad geographical distribution, although some types show regional specificity. A quarter of the sequenced R3 plasmids are associated with AMR genes, with carbapenemases being the most common. Key AMR genes include the already described *bla_OXA-58_*, *bla_OXA-72_*, *bla_OXA-24_* (carbapenem resistance), *tet39* (tetracycline resistance), *sul2* (sulfonamide resistance), and *msr-mph(E)* (macrolide resistance). Notably, despite rarely, the *mcr* gene conferring resistance to the last resort antibiotic colistin was also found [38,55].

A significant proportion of RP-type plasmids encoding RepPriCT_1 (Pfam03090) carry at least one AMR gene with RP-T1 carrying *bla_OXA-23_* (carbapenemase) and/or *aphA6* (amikacin resistance). These plasmids have been acquired by major global clones, including GC1, GC2, ST10, ST15, ST25, ST79, and ST622 [55,56].

Most of the so far sequenced plasmids lacking an identifiable replication initiation gene harbor at least one AMR gene. This group encompasses various plasmid variants, including pRAY* [57], large conjugative plasmids like pA297-3 [58], and pNDM-BK0 [59].

The small plasmid pRAY* and its variants play a key role in the spread of the *aadB* gene, which confers resistance to clinically significant antimicrobials, including tobramycin, gentamicin, and kanamycin [57]. These plasmids are widely disseminated in clinical strains from various STs, including ST1, ST81, ST2, ST25, and ST85 [55].

Conjugative plasmids encoding the MPFF transfer system form a diverse group of large plasmids found in at least 11 distinct STs, including major global clones such as ST1, ST10, and ST25 [55]. A representative example is pA297-3, which carries the *sul2* and *strAB* genes, conferring resistance to sulfonamides and streptomycin, respectively [58]. These plasmids also frequently harbor resistance genes such as *msr-mph(E)* (macrolides), *bla_PER-7_* (extended-spectrum β-lactamases), and *armA* (aminoglycosides). Notably, the *bla_NDM_* gene, responsible for carbapenem resistance, was found in two plasmids from strains isolated in India. The same gene was also identified in another group of conjugative plasmids related to pNDM-BJ01, which encode the MPFT-type conjugative transfer system [55]. These plasmids were found in strains from clinical, environmental (wastewater), and animal samples across multiple countries, highlighting their global distribution. The presence of *bla_NDM_* on conjugative plasmids in *A. baumannii* is significant, highlighting the potential for rapid transmission of this critical carbapenemase through horizontal gene transfer.

### 3.2. Resistance Islands (AbaRs)

The AbaR family of resistance islands is central to the antimicrobial resistance profiles of clones GC1 and GC2. This important class of MGEs exhibits variable genetic structural features involving different but closely related transposon backbones, diverse insertion sequences, and combinations of antibiotic-resistance genes conferring resistance to aminoglycosides, tetracycline, sulfonamides, and beta-lactams [60].

Temporal phylogenetic analyses date the emergence of resistance in GC1 to the integration of AbaR0 into the *comM* gene of a GC1 isolate during the mid-1970s [53]. This resistance island, consisting of a Tn6019 backbone and carrying genes for resistance to heavy metals and antibiotics, evolved in situ, resulting in the emergence of AbaR3 in the 1990s. The latter is distinguished by a 108 bp deletion in the *intI1* gene of the 5′-conserved segment (5′-CS) of the class 1 integron [61]. Subsequent microevolution of these islands through insertions and deletions of antimicrobial resistance genes, as well as through IS26-mediated deletions of parts of the AbaR backbone, produced many variants [53].

All GC1 lineage 1 genomes feature resistance islands with a Tn6019 backbone in the *comM* gene. This transposon includes genes for resistance to arsenate and arsenite and incorporates a large composite transposon flanked by two copies of Tn6018, which may confer cadmium and/or zinc resistance. The central region, termed the multiple antibiotic resistance region, contains antibiotic and mercuric ion resistance genes varying in length and content [62].

In contrast, lineage 2 GC1 genomes lack transposons in *comM* or feature a different element, Tn6022, which does not carry antibiotic-resistance genes. In one isolate (D36), AbaR4 was formed when a Tn2006 carrying the *oxa23* carbapenemase resistance gene, which was responsible for carbapenem resistance, was inserted into Tn6022 [63]. AbaR4 has been identified in both the chromosome and on a conjugative plasmid [64] and has been linked to carbapenem resistance in *A. baumannii* strains isolated in Australia, Republic of Korea, Taiwan, and Europe [63,65]. Notably, an *Acinetobacter* AbaR4-D36-type resistance island encoding *bla_OXA-23_* was reported in *Proteus mirabilis*, documenting the interspecies transfer of genomic islands and resistance genes [66].

In GC2, the most globally abundant clonal group resistance genes are distributed across genomic resistance islands (AbGRI1-5) located at distinct chromosomal sites. These islands have distinct transposon backbone structures and encode resistance to a broad spectrum of antibiotics, including tetracyclines (*tetA(B)* and *tetR(B)*), aminoglycosides (*aacC1*, *aacA4*, *aphA1b*, *aadA1*, *strA*, *strB*, and *armA*), sulfonamides (*sul1* and *sul2*), β-lactams (*blaTEM*), and carbapenems (*oxa23*) [67,68,69].

AbaRs can transfer between strains of the same or different sequence types through mechanisms like hitchhiking on MGEs or homologous recombination [52,60,70]. Since most *A. baumannii* isolates are competent for natural transformation, this process is expected to play a key role in horizontal gene transfer. Transformation permits the uptake and integration of large DNA fragments, including non-homologous sequences flanked by homologous regions, favoring the acquisition of MGEs that often encode advantageous traits, such as resistance to antibiotics. For example, high rates of AbaR transfer have been observed within mixed populations, underscoring the efficiency of transformation [71]. However, the fitness costs associated with MGEs have driven bacteria to use transformation not only for acquiring beneficial elements but also for excising non-advantageous ones through genome-cleansing activity [72].

Genomic analyses revealed that AbaRs are present in 66% of *A. baumannii* genomes and are mainly located in the chromosome, with *comM* being interrupted by AbaR in 96% of the cases. Additional AbaR occurrences at alternative loci or on plasmids are typically observed only when *comM* already contains an AbaR insertion. The insertion of AbaRs into *comM* appears to be a strategic adaptation to counteract the genome-cleansing effects of transformation [70]. The *comM* gene encodes a helicase that facilitates natural transformation. While not entirely inhibiting the process, its inactivation reduces bacterial transformability, allowing AbaRs to evade the genome-purging effects of transformation while still enabling recombination-mediated acquisition of beneficial mutations, such as the fluoroquinolone resistance-conferring SNPs in the *gyrA* and *parC* genes [52,70]. This strategy provides a dual benefit for *A. baumannii*: the persistence of AbaRs in the genome and the retention of adaptive flexibility to respond to environmental pressures [70].

### 3.3. Insertion Sequences (ISs)

Insertion sequences (ISs) are the smallest mobile genetic elements, consisting of terminal inverted repeats flanking one or two open reading frames encoding a transposase enzyme. When inserted into bacterial genomes, IS elements can disrupt or modify genes, influencing bacterial evolution and adaptability by introducing mutations or altering gene expression.

In *A. baumannii*, the transposition of insertion sequences (ISAba) can enhance antibiotic resistance by modifying bacterial gene expression. For instance, ISAba1 or ISAba125 elements can insert upstream of intrinsic β-lactamase genes such as *ampC* and *bla_OXA-51_*. While these genes do not confer clinical resistance at basal expression levels, IS insertion provides a strong outward promoter, leading to increased expression and resistance to third-generation cephalosporins (via *ampC*) or carbapenems (via *bla_OXA-51_*) [73].

ISAba1 insertion can also promote resistance through the overexpression of efflux pumps, which confer broad resistance to aminoglycosides, tetracyclines, β-lactams, and tigecycline. This occurs either through ISAba1 insertion upstream of *adeS*, as previously described, as part of the AdeRS two-component system that activates the AdeABC efflux pump [74], or by the ISAba1-encoded promoter driving the transcription of *adeIJK* efflux pump genes [75].

Furthermore, IS elements can contribute to resistance by disrupting genes that encode membrane or secretory proteins critical for antibiotic entry, as well as transcriptional regulators or antibiotic targets. For example, multiple studies of carbapenem-resistant *A. baumannii* isolates have revealed that ISAba825, ISAba125, ISAba10, and ISAba27 insertions disrupt the *carO* gene, which encodes an outer membrane protein essential for antibiotic uptake [76]. Transposition of ISAba elements, including ISAba1, ISAba125, and ISAba27, also disrupts *adeN*, a repressor of the AdeIJK efflux pump genes, leading to resistance against multiple antibiotics [77].

Importantly, IS elements have also been implicated in conferring resistance to colistin, a last-resort antibiotic. As already described, colistin resistance in *A. baumannii* can result from the complete absence of LPS production due to disruptions in the biosynthetic pathway and point mutations in lipid A biosynthesis genes, such as *lpxA*, *lpxC*, and *lpxD*. Moffatt et al. demonstrated that ISAba11 insertion inactivates *lpxA* or *lpxC*, leading to the loss of LPS production and resulting in colistin resistance [78]. Similarly, ISAba11 insertion in *lpxC* has been identified as a mechanism contributing to the same resistance effect [79].

Another strategy for colistin resistance involves modifications to LPS in the outer cell envelope. As described in the previous section, the addition of phosphoethanolamine (pEtN) to lipid A reduces the negative charge on the cell membrane, thus decreasing its affinity for colistin. Furthermore, Lesho et al. identified the *eptA* gene in clinical isolates of *A. baumannii*, which encodes an alternative pEtN transferase [80]. ISAba1 transposition upstream of the gene *eptA* in *A. baumannii* increased its expression and resulted in resistance to colistin [81,82]. Finally, plasmid-mediated colistin resistance has been reported and linked to the expression of the *mcr* gene, encoding a pEtN transferase involved in colistin efflux [83].

## 4. Therapies Against *A. baumannii* Infections

### 4.1. Current and Novel Antibiotic Therapies

The increasing prevalence of multidrug-resistant *A. baumannii* strains has prompted researchers to explore alternative treatment strategies. With the growing resistance to last-resort antibiotics and the risk of selection and spread of CRAB strains, synergistic antibiotic combinations offer a potential solution by enhancing the efficacy of existing therapies and reducing the insurgence of resistant strains. Currently, the most employed therapies for treating CRAB invasive infections include the administration of a dual antibiotic combination of polymyxins, tetracyclines, and β-lactams, selecting them based on their effect in vitro [84]. Unfortunately, in vitro efficacy does not always translate into good results in humans, as demonstrated in the case of colistin-meropenem combination therapy [85]. However, the use of colistin–meropenem in combination with a third antibiotic could improve the effect, particularly when ampicillin–sulbactam is included [86].

Recently approved new weapons against CRAB infections are cefiderocol (authorized by the FDA in 2019 and a few months later in the European Union by EMA) and sulbactam–durlobactam (approved by the FDA in 2023). Cefiderocol is a siderophore cephalosporin that, thanks to its very high efficacy in vitro against a wide panel of CRAB isolates [87], was proposed as a potent alternative therapy for these infections. However, the real-world evidence dampened the enthusiasm, considering the contrasting results extrapolated from clinical trials and observational studies [88]. Indeed, these underline the limitations of this therapy, including the reported insurgence of resistant strains [89], although this could be easily mitigated by using cefiderocol in combination [89]. Sulbactam–durlobactam is a β-lactam/β-lactamase inhibitor combination able to renew the sulbactam efficacy against *A. baumannii* strains expressing class D OXA carbapenemases through the next-generation β-lactamase inhibitor durlobactam. Since limited clinical data are available so far, its potential is not yet established, but data from the recently published phase III clinical trial are very encouraging [90].

Only a few antibiotics are in the clinical pipeline, and only a limited number of them have a Gram-negative spectrum of action [91]. Among these, polymyxin analogs are promising candidates for future treatments. In particular, SPR206 demonstrated improved safety and pharmacokinetics [92] and showed higher efficacy, alone and in combination, against colistin-resistant isolates carrying mutations in *lpxACD* or *pmrA* and *pmrB* genes compared with colistin [93].

Among antimicrobial compounds in the early clinical phase, a new class of antibiotics targeting the LPS transport was recently discovered, and zosurabalpin was the best candidate [94]. This molecule demonstrated high efficacy against pan-drug-resistant *A. baumannii*, overcoming the most common resistance mechanisms of this bacterium. The promising results achieved in in vivo infection models [94] and in phase I clinical trials [95] further confirmed its potential as a future treatment option.

Concerning high potential molecules in the preclinical phase, Wang and colleagues discovered and developed a naturally inspired enhanced polymyxin, macolacin, which showed high efficacy in vitro and in vivo against CR and XDR *A. baumannii* strains expressing the phosphoethanolamine transferase MCR-1 [96]. In the future, macolacin is expected to be a therapeutic weapon to combat the widespread colistin resistance.

Nowadays, high throughput in silico screening techniques are valuable tools for antibiotic discovery, as demonstrated by the work of Boulaamane et al. [97]. Specifically, they exploited artificial intelligence-based analysis of activity and pharmacological and pharmacokinetic profiles of more than 10,000 natural compounds to select molecules effectively targeting OmpW, a promising potential target in *A. baumannii* [97]. Another noteworthy example is the work by Borges and colleagues, which identified a new effective molecule by an in silico chemogenomics approach. This enabled a target homology-based identification of promising candidates among already approved drugs [98], potentially reducing the time needed for patient safety evaluation.

### 4.2. Alternative Treatments

A concrete response to the worrisome increase in MDR *A. baumannii* strains cannot involve only the research of new antibiotics as it is difficult to identify and develop them in a reasonable time.

A promising alternative approach is the use of antibiotic adjuvants, which are molecules devoid of intrinsic antibacterial activity that show potent synergy with antibiotics when used in combination and can reduce the risk of resistance selection [99]. Membrane perturbing antibiotic adjuvants (MPAAs) exert their effect on the outer membrane, generally by interacting with the LPS. This weakens the permeability barrier of the Gram-negative cell envelope, making them susceptible to the Gram-positive spectrum of antibiotics. A few interesting MPAA compounds were characterized and tested against *A. baumannii* in the last few years, including polymyxin derivatives [100], synthetic peptides [101,102], and small molecules [103]. However, the polymyxin derivative SPR741 is the only MPAA in phase I clinical trials [100], showing an adequate safety profile that will grant its further development in phase II clinical trials.

The use of bacteriophages to kill antibiotic-resistant bacteria represents another valid alternative to treat MDR *A. baumannii* infections. Indeed, phages present several key advantages: they are highly species-specific, limiting the side effects on the human microbiome, and they are not affected by antibiotic resistance mechanisms developed by bacteria. Moreover, by replicating inside bacteria, phages increase their number at the infection site without the need for multiple administrations. However, the lytic phages—most employed in phage therapy—exert a strong selective pressure on bacteria, causing the early insurgence of phage-resistant strains and the failure of the treatment. In *A. baumannii*, phage resistance is mainly mediated by mechanisms that include adsorption inhibition through the modification or the loss of the bacterial capsule or LPS (the phage main receptors) [104], the CRISPR-Cas immune system, which cleaves specific sequences of the phage genome [105], and restriction–modification (R-M) systems, consisting of methyltransferases and restriction enzymes degrading non-methylated phage DNA or RNA [106]. Phage-antibiotic combinations can be used to overcome this drawback, also because the phage-resistant phenotype is often correlated with reduced bacterial fitness and resensitization to antibiotics, generally due to mutations affecting the capsule biosynthesis [107]. For this reason, few studies have been performed in the last years to investigate this aspect. Some of them defined the efficacy of phage–antibiotic combinations against MDR and pan-drug-resistant *A. baumannii* in different in vivo models, including combinations of different phages with ceftazidime [108], ciprofloxacin [109], and meropenem [110], demonstrating an increased activity of the combination compared with the single treatments. Other works investigated the diverse, interesting aspects of phage–antibiotic synergy, such as the impact of treatment order on the efficacy and the development of the resistance to the phages [111] or the identification of molecular determinants leading to the *A. baumannii* resensitization to colistin [112] and β-lactams [107], with the final goal to set up an optimized phage-antibiotic therapy for clinical use. Bacteriophages are known to possess remarkable biofilm inhibition and eradication potential. This ability is associated with their polysaccharide hydrolases, i.e., tailspike depolymerases, that can degrade capsular polysaccharides and exopolysaccharides, exposing sessile bacteria not only to phage recognition but also to the immune system and antibiotics. This characteristic can be exploited to develop new antibiofilm treatments that involve the design of phage cocktails [113] or the use of phage-derived recombinant depolymerases as monotherapy [114] or as adjuvants in combination with antibiotics [115]. Given the important role of biofilm formation in the *A. baumannii* virulence and resistance, in addition to these treatments, additional antibiofilm therapies were described, including the use of natural compounds [116], antimicrobial peptides [117], quorum sensing inhibitors [118], already approved antihistamine drugs [119], and FtsZ protein inhibitors [120].

## 5. Conclusions

**Figure 1:**
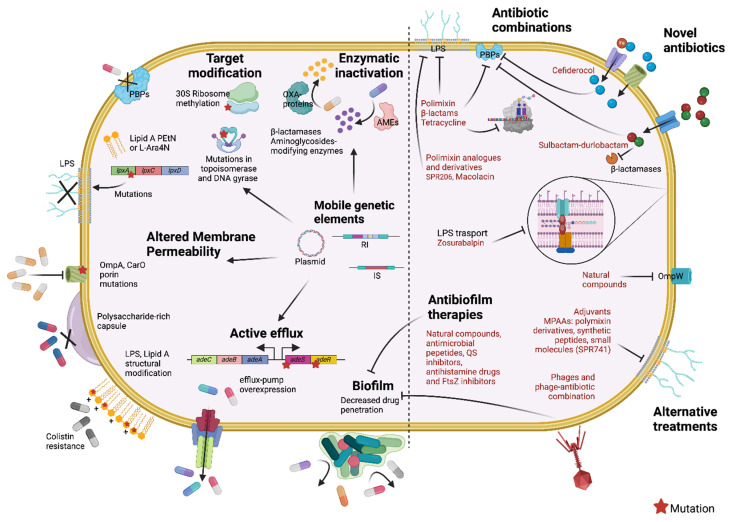
*Acinetobacter baumannii* resistance mechanisms, mobile genetic elements (left), and therapies (right). Abbreviations: *adeABC* and *adeSR*—Efflux pump genes and regulators: outer membrane protein (*adeC*), multidrug transporter (*adeB*) and membrane fusion protein (*adeA*), sensor kinase (*adeS*) and responsive regulator (*adeR*); AMEs—aminoglycoside-modifying enzymes; CarO—carbapenem resistance-associated outer membrane protein; IS—Insertion sequence; L-Ara4N—4-amino-4-deoxy-L-arabinose; LPS—lipopolysaccharide; *lpxACD*—genes codifying lipid A biosynthetic proteins; MPAAs—membrane perturbing antibiotic adjuvants; OXA type proteins—carbapenemases; OmpA—outer membrane protein A; OmpW—outer membrane protein W; PBPs—penicillin-binding proteins; PEtN—phosphoethanolamine; QS—quorum sensing; RI—Resistance islands. Created in https://BioRender.com, accessed on 7 January 2025.

In this review, we described the main mechanisms of drug resistance, their evolution, and the related consequences of therapies available so far to treat *A. baumannii* infections (Figure 1).

Enzymatic inactivation, including the production of β-lactamases and aminoglycoside-modifying enzymes, is spreading among *A. baumannii* clinical isolates. On the other hand, target site modifications due to the accumulation of mutations in genes encoding the main drug targets have been reported frequently. At the same time, altered membrane permeability and active efflux account for a reduced intracellular concentration of the above-mentioned classes of antibiotics, making the situation even worse. Moreover, the ability of *A. baumannii* to form biofilm greatly contributes to its high resistance level.

The evolution of drug resistance in this pathogen is mainly driven by the presence of mobile genetic elements, which are able to rapidly spread among different strains by horizontal gene transfer, leading to genetic diversity. Here, we described plasmid-associated resistance, which is responsible for carbapenem, tetracycline, sulfonamide, and macrolide resistance. Also, the so-called resistance islands, with a transposon backbone, insertion sequences, and drug resistance genes, have been reported as responsible for resistance to heavy metals, carbapenems, tetracyclines, aminoglycosides, sulfonamides, and β-lactams. Finally, insertion sequences have been shown to modify gene expression, contributing to drug resistance as well; in the case of β-lactams for the involvement of β-lactamase genes, for aminoglycosides, tetracyclines, β-lactams, and tigecycline for the overexpression of efflux pumps, for carbapenems for the membrane or secretory protein-encoding genes, and for colistin due to the complete lack or modification of the LPS.

In this context, new therapies are of primary importance, with the combination strategy being the most adopted so far. Moreover, the availability of new compounds, including cefiderocol (a siderophore cephalosporin), sulbactam–durlobactam (a β-lactam/β-lactamase inhibitor combination), zosurabalpin (which targets LPS transport), and macolacin (a polymyxin) is very promising. Among alternative treatments, the use of antibiotic adjuvants and bacteriophages appears to be a valid alternative to currently used antibiotics.

However, the literature has extensively demonstrated that the persistent issue of antibiotic resistance in *A. baumannii* extends beyond clinical isolates, with AMR genes having also been identified in numerous plant and animal isolates [121,122]. Environmental isolates contain virulence genes similar to those of clinical strains, potentially serving as reservoirs for resistance determinants between these distinct ecological niches [123,124]. This highlights global concerns over the evolving resistance of *A. baumannii*, emphasizing the need for state-of-the-art strategies and a transdisciplinary One Health approach for interconnecting human, animal, and environmental health [123].

## Figures and Tables

**Table 1:** Mechanisms of antibiotic resistance in *A. baumannii*.

Mechanism of Resistance	Target	Genes/Proteins	Antibiotic	Localization	Ref.
Enzymatic inactivation	Class A β-lactamases	*bla_SCO-1_*, *bla_TEM-92_*, *bla_SHV_*, *bla_GES-11_*, *bla_GES-14_*, *bla_PER-1_*, *bla_PER-7_*, and *bla_VEB-1_*	Penicillins, carbapenems	Chromosomal, plasmid, and mobile genetic elements	[27]
Enzymatic inactivation	Class B metallo-β-lactamases	*blaVIM-1*, IMP-1,IMP-2, IMP-4, IMP-5, IMP-9,IMP-10,VIM-1, VIM-2, VIM-3,VIM-4,VIM-11, SIM-1, NDM-1	Penicillins, cephalosporins, carbapenems	Plasmids and integrons	[28]
Enzymatic inactivation	Class C β-lactamases	*ampC*/AmpC	Cephalosporins, carbapenems, sulbactams	Chromosomal	[29]
Enzymatic inactivation	Class D OXA-type oxacillinase	*bla_OXA-23_*, *bla_OXA-24_*, *bla_OXA-40_*, *bla_OXA-51_*, *bla_OXA-58_*, *bla_OXA-72_*, *bla_OXA-143_* and *bla_OXA-235_*	Carbapenems	Chromosomal and plasmid	[30,31]
Enzymatic inactivation	Aminoglycoside-modifying enzymes	*aac* genes, *ant* genes, *aad* genes, *aph* genes	Aminoglycosides	Chromosomal, integron, transposon, integrative conjugative element, plasmid, chromosomal genomic island	[32,33,34]
Target site modification	Penicillin-Binding Protein (PBP)	*ftsI_A515V* and other penicillin-binding proteins PBP3	β-lactams	Chromosomal	[35]
Target site modification	16S rRNA of the 30S ribosomal subunit	*armA*, *rmtB*, *rmtB1* and *rmtE*	Aminoglycosides	Chromosomal and plasmid	[36]
Target site modification	Lipid A, LPS	*pmrCAB*, *mcr*, *hns-eptA*, *lpxA*, *lpxC* and *lpxD*	Colistin	Chromosomal and plasmid	[37,38,39,40,41]
Target site modification	DNA gyrase and topoisomerase IV	*gyrA* and *parC*	Fluoroquinolones	Chromosomal	[42]
Altered membrane permeability	Porins	*ompA*, *carO*	β-lactams, aminoglycosides, tigecycline, carbapenems	Chromosomal	[43]
Altered membrane permeability	LPS	*lpsB*, *lptD*, and *vacJ*	Polymyxins, colistin	Chromosomal	[44]
Altered membrane permeability	polysaccharide-rich capsule	capsule biosynthesis and regulatory genes	Aminoglycosides	Chromosomal	[45]
Active efflux	RND-family efflux pumps and MATE-family efflux pumps	*adeABC*, *adeRS*, *adeFGH*, *adeIJK*, *abeM*, and *qepA*	Aminoglycoside, carbapenems, fluoroquinolones, cephalosporins, chloramphenicol, erythromycin, tetracycline, and tigecycline	Chromosomal and plasmid	[43,46,47,48]
Other	Biofilm	*bap*, *ompA*, *csuE*, *pgaB,* and AbaI/AbaR quorum sensing genes	Persistence and multi-drug resistance	Chromosomal	[49,50]

## Data Availability

No new data were created or analyzed in this study.
